# Magnetic Resonance Spectroscopy in the Ventral Tegmental Area Distinguishes Responders to Suvorexant Prior to Treatment: A 4-Week Prospective Cohort Study

**DOI:** 10.3389/fpsyt.2021.714376

**Published:** 2021-08-23

**Authors:** Muneto Izuhara, Shoko Miura, Koji Otsuki, Michiharu Nagahama, Maiko Hayashida, Sadayuki Hashioka, Hiroya Asou, Hajime Kitagaki, Masatoshi Inagaki

**Affiliations:** ^1^Department of Clinical Laboratory, National Institute of Mental Health, National Center of Neurology and Psychiatry, Tokyo, Japan; ^2^Department of Sleep-Wake Disorders, National Institute of Mental Health, National Center of Neurology and Psychiatry, Tokyo, Japan; ^3^Department of Psychiatry, Faculty of Medicine, Shimane University, Izumo, Japan; ^4^Department of Radiology, Faculty of Medicine, Shimane University, Izumo, Japan

**Keywords:** magnetic resonance spectroscopy, insomnia, suvorexant, orexin, glia, ventral tegmental area, psychiatric patients

## Abstract

**Background:** The ventral tegmental area (VTA; a dopaminergic nucleus) plays an important role in the sleep-wake regulation system including orexin system. In addition to neuronal activity, there is increasing evidence for an important role of glial cells (i.e., astrocytes and microglia) in these systems. The present study examined the utility of magnetic resonance spectroscopy (MRS) for detecting neural and/or glial changes in the VTA to distinguish responders from non-responders before treatment with the orexin receptor antagonist suvorexant.

**Methods:** A total of 50 patients were screened and 9 patients were excluded. The remaining 41 patients with insomnia who have or not a psychiatric disease who were expected to receive suvorexant treatment were included in this study. We compared MRS signals in the VTA between responders to suvorexant and non-responders before suvorexant use. Based on previous reports, suvorexant responders were defined as patients who improved ≥3 points on the Pittsburgh Sleep Quality Index after 4 weeks of suvorexant use. MRS data included choline (reflects non-specific cell membrane breakdown, including of glial cells) and N-acetylaspartate (a decrease reflects neuronal degeneration).

**Results:** Among 41 examined patients, 20 patients responded to suvorexant and 21 patients did not. By MRS, the choline/creatine and phosphorylcreatine ratio in the VTA was significantly high in non-responders compared with responders (*p* = 0.039) before suvorexant treatment. There was no difference in the N-acetylaspartate/creatine and phosphorylcreatine ratio (*p* = 0.297) between the two groups.

**Conclusions:** Changes in glial viability in the VTA might be used to distinguish responders to suvorexant from non-responders before starting treatment. These findings may help with more appropriate selection of patients for suvorexant treatment in clinical practice. Further, we provide novel possible evidence for a relationship between glial changes in the VTA and the orexin system, which may aid in the development of new hypnotics focusing on the VTA and/or glial cells.

## Introduction

An orexin receptor antagonist was recently developed for clinical use to promote sleep (1). Orexin neurons promote wakefulness via excitation of brainstem nuclei including the ventral tegmental area (VTA) (2). Recent studies have also emphasized the importance of dopaminergic activity in the VTA on sleep-wake regulation (3). For example, electroencephalogram detected firing pattern changed across sleep-wake cycle, or optogenetic or chemogenic stimulation of the VTA was found to initiate and maintain wakefulness (4, 5).

There is accumulating evidence for an important role of glial cells in controlling the orexin system and sleep-wake regulation, in addition to the roles of neurons in sleep disturbances in various neurodegenerative disorders (6). For example, morphological changes of astrocytes can result in decreased concentrations of extracellular adenosine, an important sleep promoting molecule (7). Pharmacological inhibition of microglial activity was also reported to reduce the compensatory increase in slow wave activity following sleep deprivation (8). Further, the orexin system can regulate inflammation and associated insomnia via control of microglial reactivity (9). Finally, astrocytes were found to convey orexin activity to remote brain areas utilizing a local network (10).

In the present study, we examined the hypothesis that differences in neuronal and/or glial cell function in the VTA are important for the responses of patients to the orexin receptor antagonist suvorexant and that these differences can be detected by magnetic resonance spectroscopy (MRS). MRS is a technique that measures metabolite changes related to neuronal or glial cell viability (11). To our knowledge, no studies using MRS have examined the brainstem in patients with insomnia. We conducted a 4-week cohort study of patients with insomnia to examine whether MRS findings can distinguish responders from non-responders before starting suvorexant treatment.

## Methods

### Ethics Statement

The present study was approved by the ethics committee of Shimane University Hospital (Shimane, Japan) (Approval No: 20170814-1) in August 2017. Detailed information on the aims and protocols of the study were explained to the participant. The participants provided their written informed consent in this study.

### Participants

This 4-week cohort study was conducted in the Psychiatry Department of Shimane University Hospital from October 2017 to February 2019. Consecutive patients with insomnia symptoms were enrolled using the following inclusion criteria: (a) met the diagnostic criteria of insomnia regardless of comorbid psychiatric disease; (b) were expected to be treated with suvorexant for at least 4 weeks and did not use medicines that are prohibited to combine with suvorexant; (c) were intended to receive magnetic resonance imaging (MRI) examination to detect and/or exclude organic diseases; (d) agreed with the extended MRS examination in addition to routine MRI; and (e) who or whose caregivers could answer a sleep questionnaire. Patients were included in this study regardless of the type of primary disease. We excluded patients based on the following exclusion criteria: (a) in a coma; (b) were strongly sedated with anesthetics; (c) were allergic to suvorexant; (d) had a history of drug abuse or drug dependency; (e) were pregnant or were planning pregnancy; (f) were breastfeeding (suvorexant has not been proven to be safe in infants); (g) could not swallow the suvorexant pill; or (h) participated in another trial within 3 months before entering this study. We recruited patients in acute psychiatric ward. Therefore, almost all patients were in acute phase of psychiatric disease. Patients were diagnosed using Diagnostic criteria of Mental Disease 5th edition (DSM-5) by specialized psychiatrists.

### Measurements

All patients were questioned with the Pittsburgh Sleep Quality Index (PSQI) (12) before and after 4 weeks of suvorexant use. The PSQI measures sleep quality and quantity based on recall of sleep behaviors over the prior 4-week period. The 19 PSQI questions measure seven categories of sleep, including quality, latency, duration, efficacy, disturbances, hypnotic use, and daytime dysfunction. Domain scores are coded from 0 to 3 and a global PSQI score is obtained by summing across the domains. The global PSQI score ranges from 0 to 21 and a score >5 is considered to reflect a patient with low sleep quality. The sensitivity and specificity of Japanese version of the PSQI estimates 85.7 and 86.6% of primary insomnia, and Cronbach's alpha is 0.77 (13).

We set the observational period as 4 weeks because a previous trial using suvorexant showed sleep improvement within this timeframe (14) and as the PSQI measures symptoms of insomnia over 4 weeks. We defined a responder to suvorexant as a patient who showed a ≥3 PSQI global score decrease, as previously reported (15, 16). The background (including gender, age, body mass index, Eastern Cooperative Oncology Group (ECOG) performance status (17), Charlson comorbid index (18), and current smoking and alcohol consumption), psychiatric disorders (DSM-5), and medication use (antidepressants, antiepileptics, antipsychotics, and hypnotics including gamma-aminobutyric acid receptor agonists and ramelteon) of the patients were also evaluated to examine for confounding factors.

### MRS

MRS can be used to determine the concentration of metabolites in the brain (11) including choline [which reflects non-specific cellular membrane breakdown (19)], creatine and phosphorylcreatine [the level of creatine and phosphorylcreatine is constant and is used as a reference value (20)], N-acetylaspartate [NAA; an NAA signal decrease reflects neuronal and/or myelin degeneration (21)], and myo-inositol [a marker of microglial activation (22)]. Participants were scanned using a PRESS (point-resolved spectral sequencing) sequence with a repetition time (TR)/echo time (TE) of 1,500 ms/35 ms, respectively.

**Figure 1:**
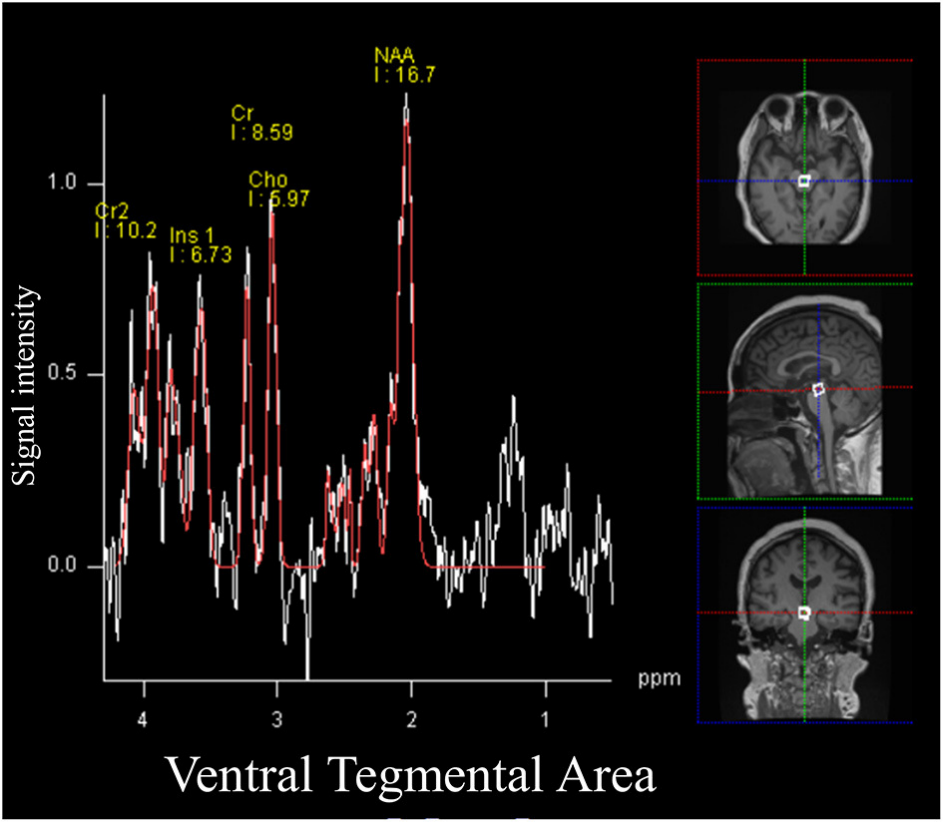
Magnetic resonance spectroscopy (MRS) signals and the volume of interest for the ventral tegmental area. All participants underwent a single voxel point-resolved spectral sequencing scan. The voxel size was set as 1.0 × 1.0 × 1.0 cm.

The spectral acquisition was applied to the VTA. A region of interest was set for the VTA using a voxel size of 10 × 10 × 10 mm, while 133 frames were collected for all participants (Figure 1) (23). Other orexin projecting areas including the locus coeruleus (13 mm^3^) (24), dorsal raphe nuclei (70 mm^3^) (25), laterodorsal/pedunculopontine tegmental nuclei (100 mm^3^) (26), and the tuberomammillary nucleus (in the posterior hypothalamus; 130 mm^3^) (27) were too small to allow for signal acquisition compared with the VTA (360 mm^3^) (28). MRS data were analyzed using commercial software (Siemens Syngo VE11C; Neuro 3D, Siemens, Erlangen, Germany) (29). To ascertain the adequate signal-noise ratio, the attending MRI specialized clinical technician checked full width at half maximum (FWHM) every MRS examination and confirmed FWHM <20 Hz.

### MRI Acquisition

MRI was obtained on a 3.0T MAGNETOM PRISMA system (Siemens) using a 20-channel phased array coil. For conventional MRI scans, the parameters for T1-weighted imaging were TR/TE = 410 ms/13 ms, respectively, and field of view (FOV) = 22 cm × 22 cm, the parameters for T2-weighted imaging were TR/TE = 3400 ms/100 ms, respectively, and FOV = 22 × 22 cm, and the parameters for T2-fluid-attenuated inversion recovery imaging were TR/TE = 12,000 ms/130 ms, respectively, and FOV = 22 × 22 cm. Additional MRI sequences included three-dimensional T1 magnetization-prepared rapid gradient echo (TR = 2,300 ms, TE = 2.4 ms, inversion time = 991 ms, bandwidth = 200 Hz, number of averages = 1, matrix size = 154 × 256, coronal section thickness = 1.2 mm, flip angle = 8°, acquisition time = 3:23 min).

### Explanatory Examination

To assess the influence of diseases comorbid with insomnia, such as schizophrenia, depression, and neurocognitive disorders, on the choline/creatine and phosphorylcreatine ratio in the VTA, we compared the choline/creatine and phosphorylcreatine ratio between responders and non-responders stratified by each diagnostic criteria of comorbid psychiatric disease. To evaluate the association between insomnia severity and the choline/creatine and phosphorylcreatine ratio in the VTA, we constructed a scatter plot of the baseline PSQI global score and the choline/creatine and phosphorylcreatine ratio in the VTA.

### Statistical Analysis

A Mann-Whitney *U* test was used to compare differences in MRS signals, including the choline/creatine and phosphorylcreatine ratio and the NAA/creatine and phosphorylcreatine ratio, between suvorexant responders and non-responders. Other signals including myo-inositol are too weak to detect differences in small cohorts of patients. In this preliminary study, differences in myo-inositol signals between suvorexant responders and non-responders were examined using the Mann–Whitney *U* test for explanatory purposes. The correlations between MRS signals and difference in PSQI global score before and after 4-week of suvorexant treatment, and PSQI global score before the treatment were calculated using Spearman's test for explanatory purposes. The background data of the patients are presented as mean ± standard deviation. The Chi-square test and the Kruskal-Wallis test were used to compare the background data. The Kruskal–Wallis test was performed to compare drug use between suvorexant responders and non-responders before and after the 4 weeks of suvorexant treatment. These data are presented as median (interquartile range). A power analysis with 90% power indicated that a sample size of *N* = 20 was required, assuming an effect size of *d* = 1.0, based on previous studies using MRS in the brainstem (30, 31). All statistical analyses were performed using statistical software (SPSS v23; IBM, Tokyo, Japan). Significance was set at *p* = 0.05.

## Results

**Figure 2:**
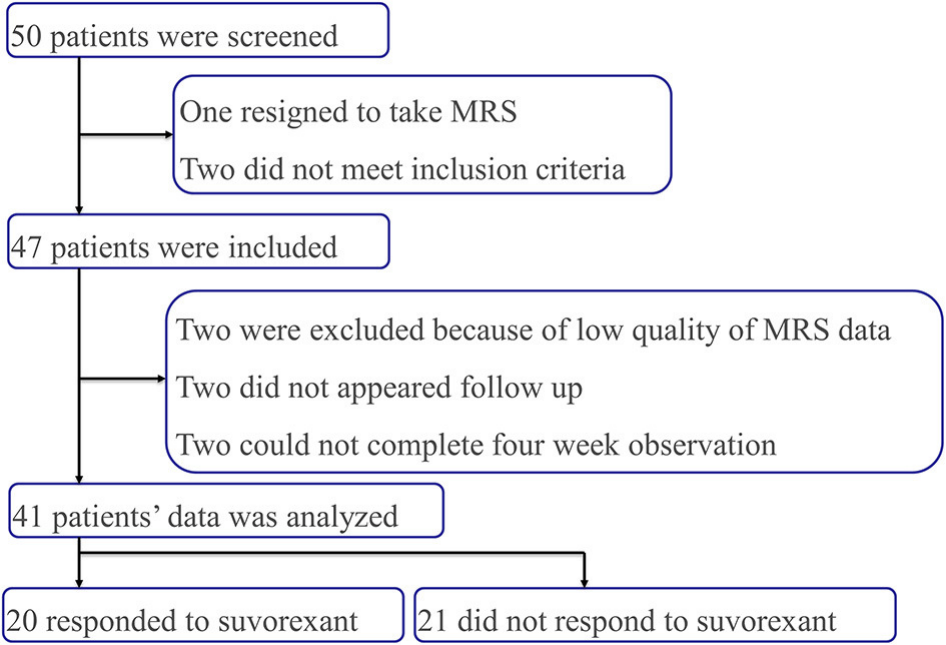
Flow chart of the study inclusion and exclusion process.

A total of 50 patients were recruited into this study (Figure 2). One patient withdrew consent to receive an MRI scan and two patients did not meet the inclusion criteria (one did not fulfill insomnia diagnostic criteria and one started clarithromycin [a cytochrome P450, family 3, subfamily A, competitive inhibitor], which is prohibited for use with suvorexant). Thus, 47 patients were included in this study. However, six more patients were excluded; MRS data could not be used for two patients (one patient could not stop moving during MRS examination and one patient exhibited iron deposition near the globus pallidus that disturbed the MRS signals), two patients did not attend the follow-up examination, and two patients did not complete the 4-week observation period (one patient developed liver failure and one stopped taking suvorexant because of somnolence during the daytime and could not complete the follow-up questionnaire). Thus, a total of 41 patients were included in the final analysis. Of these 41 patients, 20 patients responded to suvorexant and 21 patients did not.

Suvorexant responders showed trends toward being younger (54.7 ± 13.9 years vs. 61.1 ± 14.3 years, respectively) and having a higher body mass index (24.1 ± 7.6 vs. 21.7 ± 4.3, respectively), a lower Charlson comorbid index (0.4 ± 0.7 vs. 1.0 ± 1.2, respectively), and a higher PSQI score before suvorexant use (15.0 ± 2.7 vs. 11.1 ± 4.8, respectively) compared with non-responders (Table 1). The PSQI global score was increased (worsened) among non-responders during the observational period. As shown in Supplementary Table 1, component 1(Subjective sleep quality), component 2 (Sleep latency), component 3 (Sleep duration), and component 4 (Habitual sleep efficiency) were increased. The diseases of the patients according to the DSM-5 are shown in Table 2. Patients with depression were more prevalent in suvorexant responders compared with non-responders. There were seven patients with schizophrenia and schizoaffective disorders in the responders group and six patients in the non-responders group. There were three patients with dementia in the responders group and four patients in the non-responders group. Medication use in responders and non-responders before and after the 4-week suvorexant treatment is shown in Table 3 (median number of drug use for each patient) and Supplementary Table 2 (the number of patients who use each drug). There were no differences in the number of antidepressants, antiepileptics, antipsychotics, or gamma-aminobutyric acid receptor agonists used between the responders and non-responders.

**Table 1:** Comparison of the background of the patients for responders to suvorexant and non-responders.

**Background of the patients**	**Suvorexant responders (** ***N*** **= 20)**	**Suvorexant non-responders (** ***N*** **= 21)**	***p***
No. of woman (%)	12 (60.0%)	8 (38.1%)	0.138
Age in years, average (SD)	54.7 (13.9)	61.1 (14.3)	0.230
Height in cm, average (SD)	159.5 (8.5)	161.8 (10.2)	0.361
Weight in kg, average (SD)	62.1 (22.4)	57.2 (13.0)	0.611
BMI, average (SD)	24.1 (7.6)	21.7 (4.3)	0.294
Performance status (ECOG) (17)			0.664
No. of patients scored as 0 (%)	12 (60.0%)	11 (52.4%)	
No. of patients scored as 1 (%)	3 (15.0%)	4 (19.0%)	
No. of patients scored as 2 (%)	1 (5.0%)	2 (9.5%)	
No. of patients scored as 3 (%)	4 (20.0%)	3 (14.3%)	
No. of patients scored as 4 (%)	0 (0.0%)	1 (4.8%)	
Charlson comorbid index (18), average (SD)	0.4 (0.7)	1.0 (1.2)	0.064
No. of patients with myocardial infarction (%)	0 (0.0%)	2 (9.5%)	
No. of patients with congestive heart failure (%)	0 (0.0%)	1 (4.8%)	
No. of patients with cerebrovascular accident/TIA (%)	1 (5.0%)	2 (9.5%)	
No. of patients with dementia (%)	3 (15.0%)	4 (19.0%)	
No. of patients with connective tissue disease (%)	0 (0.0%)	2 (9.5%)	
No. of patients with DM without any end-organ damage (%)	2 (10.0%)	7 (33.3%)	
No. of patients with hemiplegia (%)	1 (5.0%)	2 (9.5%)	
No. of patients with solid tumor (%)	0 (0.0%)	2 (9.5%)	
No. of current smokers (%)	0 (0.0%)	0 (0.0%)	
No. of current drinkers (%)	0 (0.0%)	0 (0.0%)	
PSQI, average (SD)			
Before suvorexant use	15.0 (2.7)	11.1 (4.8)	0.003
After suvorexant use	9.4 (2.8)	13.1 (3.9)	0.002

*SD, standard deviation; BMI, body mass index; ECOG, Eastern Cooperative Oncology Group; TIA, transient ischemic attack; COPD, chronic obstructive pulmonary disease; DM, diabetes mellitus; AIDS, acquired immunodeficiency syndrome; PSQI, Pittsburgh Sleep Quality Index*.

**Table 2:** Psychiatric disease comorbid with insomnia diagnosed by the Diagnostic and Statistical Manual of Mental Disorders-5 criteria.

**Diagnostic criteria**	**Suvorexant responders** **(** ***N*** **= 20)**	**Suvorexant non-responders** **(** ***N*** **= 21)**
Nothing (insomnia only)	1 (5.0%)	2 (9.5%)
Behcet's Disease	0 (0.0%)	1 (4.8%)
Substance/medication-induced sleep disorder insomnia type	0 (0.0%)	1 (4.8%)
Schizophrenia	7 (35.0%)	4 (19.0%)
Schizoaffective disorder	0 (0.0%)	2 (9.5%)
Delusional disorder persecutory type	0 (0.0%)	2 (9.5%)
Major depression	5 (25.0%)	0 (0.0%)
Bipolar disorder	1 (5.0%)	2 (9.5%)
Persistent depressive disorder	1 (5.0%)	1 (4.8%)
Anxiety disorder	1 (5.0%)	1 (4.8%)
Bulimia nervosa	1 (5.0%)	0 (0.0%)
Wernicke–Korsakoff syndrome	0 (0.0%)	1 (4.8%)
Frontotemporal lobar degeneration	1 (5.0%)	0 (0.0%)
Dementia with Lewy bodies	2 (10.0%)	1 (4.8%)
Alzheimer's disease	0 (0.0%)	3 (14.3%)

**Table 3:** The median number of drugs for each patient use.

**Drug use**	**Before suvorexant use**		**Four weeks after suvorexant**		**Kruskal–Wallis test**
	**Suvorexant responders**	**Suvorexant non-responders**	**Suvorexant responders**	**Suvorexant non-responders**	
Number of drugs	10.0 (6.3–17.8)	7.0 (2.0–14.0)	8.0 (5.0–12.8)	6.0 (0.0–10.0)	0.193
Number of antidepressants	0.0 (0.0–1.0)	0.0 (0.0–0.0)	0.0 (0.0–1.0)	0.0 (0.0–0.0)	0.204
Number of antiepileptics	0.0 (0.0–1.0)	0.0 (0.0–1.0)	0.0 (0.0–1.0)	0.0 (0.0–0.5)	0.760
Number of antipsychotics	1.0 (0.0–2.8)	1.0 (0.0–2.0)	1.0 (0.0–2.0)	1.0 (0.0–1.0)	0.580
Number of GABAergics	1.0 (0.0–2.0)	1.0 (0.0–1.0)	0.0 (0.0–1.0)	0.0 (0.0–1.0)	0.052
Number of Ramelteon	0.0 (0.0–0.0)	0.0 (0.0–0.0)	0.0 (0.0–0.0)	0.0 (0.0–0.5)	0.673

*For medication use, there were no significant differences in the number of medications, antidepressants (amitriptyline, mianserin, trazodone, fluvoxamine, mirtazapine, duloxetine, escitalopram), antiepileptics (phenytoin, fosphenytoin, carbamazepine, clonazepam, valproate, topiramate, lamotrigine, levetiracetam), antipsychotics (chlorpromazine, perphenazine, levomepromazine, haloperidol, zotepine, risperidone, quetiapine, perospirone, olanzapine, aripiprazole, blonanserin, clozapine, paliperidone, asenapine, brexpiprazole), or gamma-aminobutyric acid (GABA) receptor agonists (GABAnergics; estazolam, nitrazepam, flunitrazepam, brotizolamm, diazepam, lorazepam, tofisopam, loflazepate, flunitrazepam, midazolam, rilmazafone, tandospirone, zolpidem, eszopiclone, etizolam) between suvorexant responders and non-responders before or at 4 weeks after suvorexant use. Data are presented as median (25th−75th percentiles)*.

**Figure 3:**
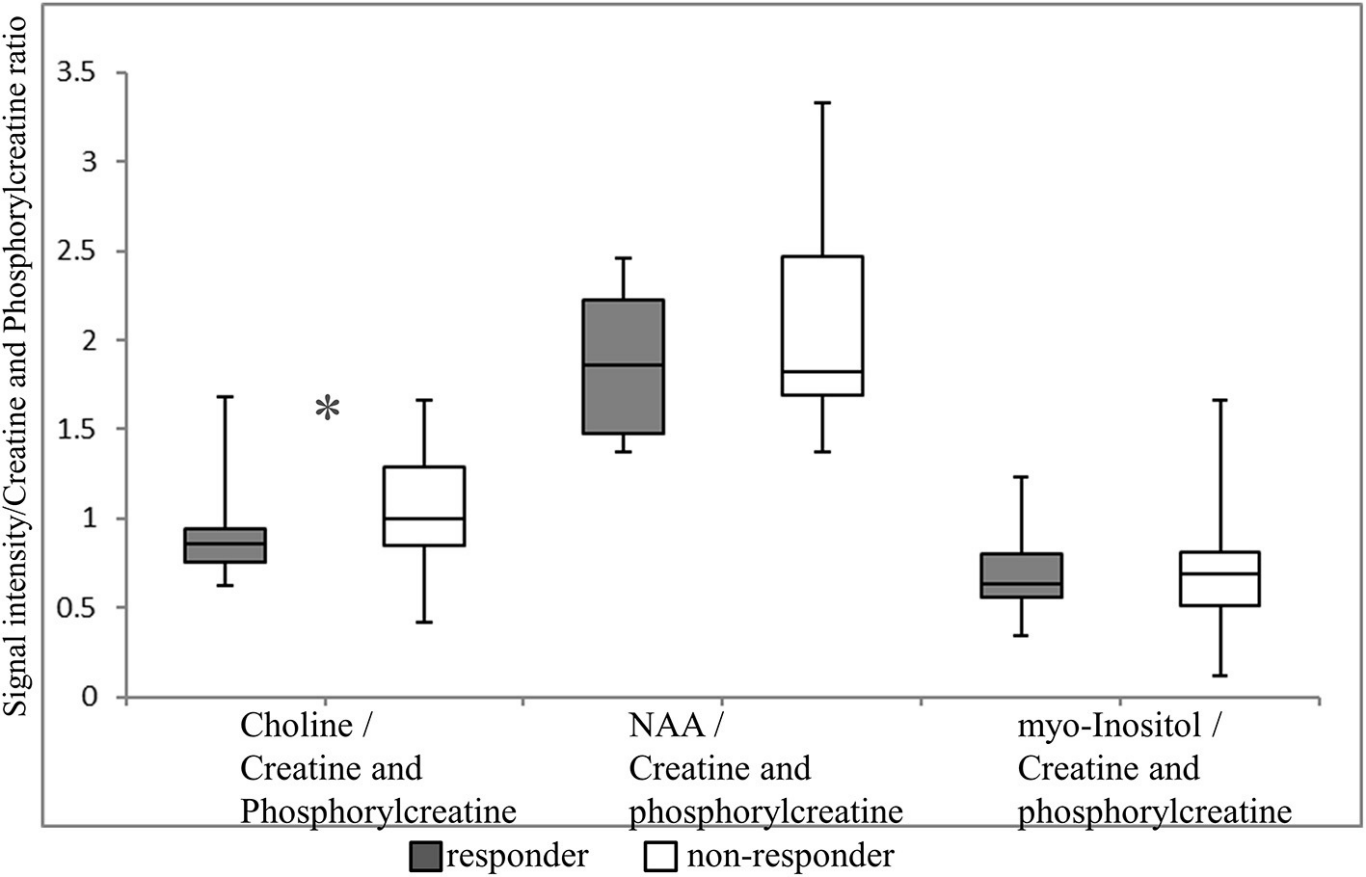
Magnetic resonance spectroscopy (MRS) signals in the ventral tegmental area. Each MRS signal is shown as a box plot. The upper and lower whiskers represent the maximum and minimum ratios, respectively. The upper, middle, and lower bars represent the 75th percentiles, median, and 25th percentiles, respectively. *p = 0.039. NAA, N-acetylaspartate.

The primary outcome of our study was that the choline/creatine and phosphorylcreatine ratio in the VTA was significantly lower in suvorexant responders (0.85; median, 0.76–0.95 for the 25th−75th percentiles) compared with non-responders (1.00; median, 0.85–1.29 for 25th−75th percentile; *p* = 0.039) (Figure 3). There were no differences in the NAA/creatine and phosphorylcreatine or the myo-inositol/creatine and phosphorylcreatine ratio between responders and non-responders (NAA/creatine and phosphorylcreatine: 1.86 [1.48–2.23] vs. 1.83 [1.69–2.47], respectively, *p* = 0.297; myo-inositol/creatine and phosphorylcreatine: 0.633 [0.56–3.90] vs. 0.681 [0.51–0.90], respectively, *p* = 0.979).

The choline/creatine and phosphorylcreatine ratios in the VTA between responders and non-responders stratified by each diagnostic criteria of comorbid psychiatric disease are shown in Table 4. The majority of the subcategories of comorbid psychiatric disease in the suvorexant responders group showed a lower choline/creatine and phosphorylcreatine ratio. Furthermore, most of the subcategories in the non-responders group showed a higher choline/creatine and phosphorylcreatine ratio.

**Table 4:** The choline/creatine and phosphorylcreatine ratio in the ventral tegmental area between responders and non-responders stratified by each diagnostic criteria of comorbid psychiatric disease.

**Choline/creatine and phosphorylcreatine ratio diagnostic criteria**	**Suvorexant responders**			**Suvorexant non-responders**		
	**Mean**	**SD**	***N***	**Mean**	**SD**	***N***
Sleep–wake disorders	0.87	-	1	1.11	0.31	4
Insomnia	0.87	-	1	1.31	0.33	2
Insomnia with Behcet's Disease	-	-	0	0.84	-	1
Substance/medication-induced sleep disorder insomnia type	-	-	0	0.99	-	1
Schizophrenia spectrum	0.91	0.35	7	1.00	0.38	8
Schizophrenia	0.91	0.35	7	1.02	0.55	4
Schizoaffective disorder	-	-	0	1.14	0.00	2
Delusional disorder	-	-	0	0.83	0.04	2
Bipolar and depressive disorders	0.86	0.11	6	1.25	0.38	5
Major depression	0.89	0.12	4	-	-	0
Bipolar disorder	0.81	-	1	1.33	0.47	2
Persistent depressive disorder	0.70	-	1	1.29	-	1
Illness anxiety disorder	0.86	-	1	1.14	0.55	2
Neurocognitive Disorders	0.95	0.33	5	0.94	0.07	4
Wernicke–Korsakoff syndrome	-	-	0	0.96	-	1
Frontotemporal lobar degeneration	1.52	-	1	1.02	-	1
Dementia with Lewy bodies	0.83	0.07	3	-	-	0
Alzheimer's disease			0	0.88	0.04	2
Bulimia nervosa	0.97	-	1	-	-	0
All	0.91	0.26	20	1.07	0.33	21

*SD, standard deviation*.

**Figure 4:**
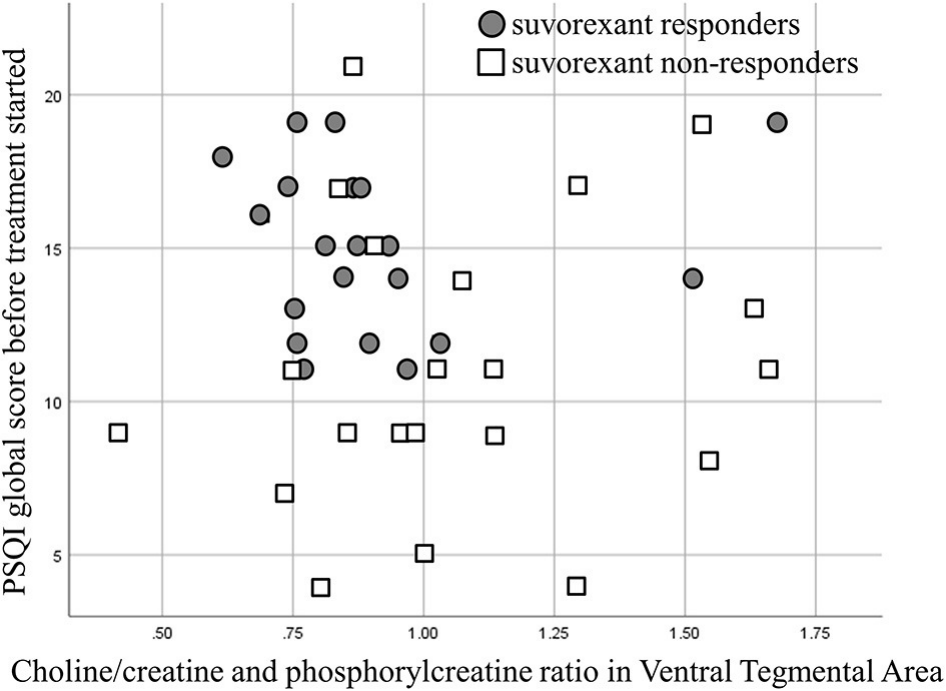
A scatter plot showing the baseline Pittsburgh Sleep Quality Index (PSQI) global score and the choline/creatine and phosphorylcreatine ratio.

A scatter plot of the baseline PSQI global score and the choline/creatine and phosphorylcreatine ratio in the VTA is shown in Figure 4. The average baseline PSQI global score of non-responders was low compared with suvorexant responders. However, the baseline PSQI global scores of non-responders were widely distributed, with some patients having high baseline PSQI scores.

**Figure 5:**
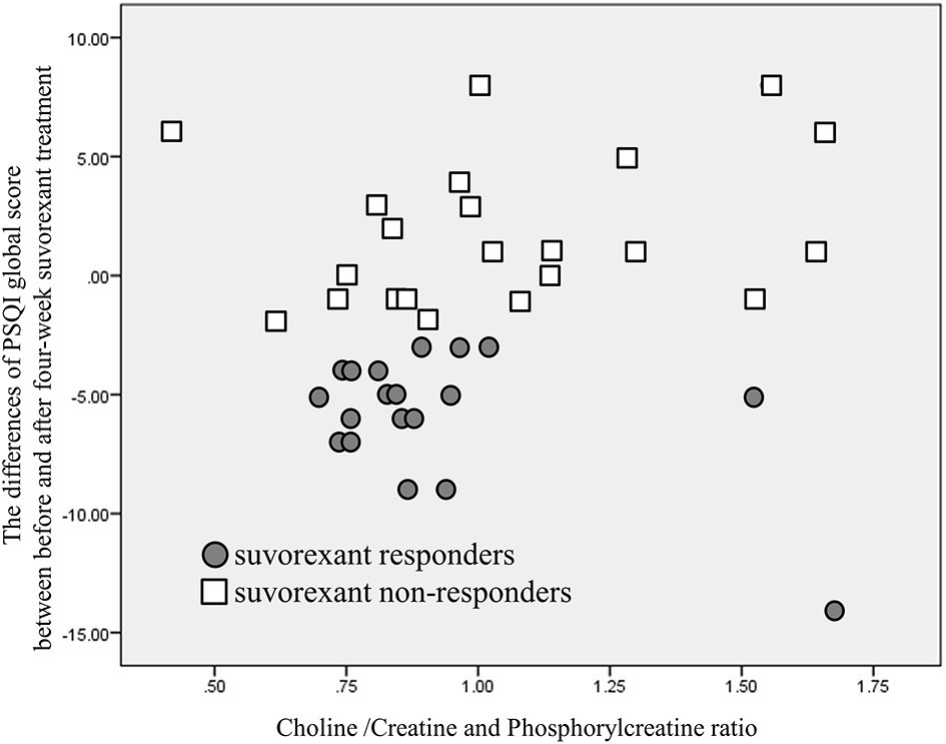
A scatter plot of choline/creatine and phosphorylcreatine ratio and the differences in PSQI global score before and after 4-week of suvorexant treatment. Although there is a tendency that lower choline/creatine and phosphorylcreatine ratio may relate to a greater decrease of PSQI global score, there is no significant correlation of choline /creatine and phosphorylcreatine ratio and the differences of PSQI global score (*p* = 0.064).

Another scatter plot of the choline/creatine and phosphorylcreatine ratio in the VTA and the differences in PSQI global score before and after 4-week of suvorexant treatment is shown in Figure 5. The correlation was not significant (correlation coefficient was 0.292, *p* = 0.064). The correlation of the choline/creatine and phosphorylcreatine ratio in the VTA and the PSQI global score before the treatment was not significant (correlation coefficient was −0.0112, *p* = 0.487).

## Discussion

A key finding of the present study was that signal changes in the VTA detected by MRS might be used to distinguish responders to suvorexant from non-responders in patients with insomnia before starting treatment. Specifically, patients with relatively high choline/creatine and phosphorylcreatine signal ratio in the VTA may show a poor response to suvorexant.

Although we observed relatively high cholinergic signal in suvorexant non-responders, there were no differences in NAA between the two groups. Relatively high cholinergic signal was reported to reflect non-specific cell membrane breakdown (19), while a decreased NAA signal reflects neuronal and/or myelinic degeneration (21). As such, our data suggest that preservation of non-neural cells such as glia may be associated with the response to suvorexant. Although previous studies have emphasized the importance of glia in sleep-wake regulation (7–10), to our knowledge there are no reports examining the relationship between glial changes and the effect of suvorexant. It is also possible that MRS differences in the choline/creatine and phosphorylcreatine ratio may reflect differences in cellular states. Future studies examining the relationship between specific changes in glia and suvorexant function may provide a further understanding of the mechanisms underlying suvorexant resistance.

In the present study, signal changes in the VTA detected by MRS may predict the response to suvorexant. The VTA plays a key role in sleep-wake regulation (4, 5), while several studies have examined the role of glia in the VTA (32, 33) and the role of the VTA in sleep (34). Furthermore, recent animal studies showed suvorexant can restore stress-induced aberrant dopamine system activity in VTA (35). However, to our knowledge, there are no reports of a direct relationship between glial changes in the VTA and sleep function. Our findings may suggest a potential connection between glial function in the VTA and the effect of suvorexant. Thus, the development of drugs to prevent glial changes in the VTA may be useful for patients who are resistant to suvorexant.

The findings from the present study suggest that it may be possible to distinguish suvorexant responders from non-responders before suvorexant treatment is started. Previous studies have examined the predictive factors for insomnia treatment, although not for the prediction of treatment prognosis using suvorexant. Further, there are inconsistent findings in these studies, including the severity of insomnia (36, 37), sleep duration (38, 39), and comorbid psychiatric disease (40, 41). Further studies examining the accuracy of prediction of suvorexant treatment may help clinicians in the selection of optimal therapy based on prognosis prediction.

The present study has several potential limitations. First, we collected patients in an acute psychiatry ward and almost all patients had comorbid psychiatric diseases. The majority of subcategories of comorbid psychiatric disease in suvorexant responders showed a relatively low choline/creatine and phosphorylcreatine ratio, whereas most of the subcategories of comorbid psychiatric disease in non-responders showed a relatively high choline/creatine and phosphorylcreatine ratio (Table 4), as same as the primary outcome of the present study. However, because of the limited number of patients in each diagnostic group, a contribution of comorbid psychiatric diseases to our findings remains possible. Furthermore, this background diversity of our patients may limit the relevance of our findings to chronic insomnia patients without any psychiatric comorbidity. In addition, psychiatric disease and stress also affect glial activity (42, 43). The present study could not detect the causal relationship between suvorexant response and psychiatric stress. Furthermore, in the present study, we could not detect the significant association between choline/creatinine and phosphorylcreatine ratio and the differences in PSQI global score before and after 4-week of suvorexant treatment, although there is a tendency that lower ratio of choline/creatinine and phosphorylcreatine ratio may be correlated with a greater decrease of PSQI global score, as shown in Figure 5. A larger sample of participants may confirm the correlation of choline/creatinine and phosphorylcreatine ratio signal and status of symptoms. Second, this study was not randomized. Therefore, the backgrounds of the patients were different between suvorexant responders and non-responders. For example, the baseline PSQI was higher in the suvorexant responder group. Although some of the non-responders had a high baseline PSQI global score (Figure 4), a randomized control study is needed to eliminate the effect of differences in baseline characteristics. Moreover, as shown in Table 3, the number of drug use was not significantly different between responders and non-responders and before and after treatment. However, there is a possibility that other drugs may modify the results. Furthermore, the assessors of the PSQI and MRS data were not blinded to the treatment groups. Thus, large blinded studies are required to eliminate these potential biases. Third, although it is important that which psychiatric disease is severely affected by degenerative cells in VTA, the limited number of participants in the present study prevents conclusive discussion. The present study cannot address the differences by diagnosis, nor can it address the association between the orexin system and the prognosis of suvorexant treatment by diagnosis. The present study warrants the need for future study with a larger number of subjects. Fourth, we did not use the linear combination model (44), which may have reduced the accuracy of our findings. During the study period, the linear combination model was not freely available. Therefore, we planned to use Siemens Syngo VE11C; Neuro 3D. From 16th February 2021, the linear combination model has become open access. Future studies should use the linear combination model. Fifth, in the present study, objective sleep measurements, such as polysomnography or actigraphy were not used. Further studies using objective sleep measurement are needed. Sixth, the size of the region of interest may have been too small to eliminate noise. As such, we also examined signals other than choline and NAA for explanatory purposes. Sixth, the size of the region of interest was 2.7 times larger than the VTA. The VTA is surrounded by nuclei such as the substantia nigra, which is another major source of dopaminergic neurons (45). Thus, further studies are required to separate the role of VTA from other dopaminergic nuclei. Finally, suvorexant is currently available only in United States, Canada, Australia, Russia, and Japan, and is not approved in other industrialized countries. And a lower dosage was approved, while the clinical trials examined the high dosage of 40 mg (46, 47). Modest effect and next day somnolence may prevent the usage of suvorexant worldwide.

## Conclusion

We found that changes in cholinergic signals in the VTA may be useful for distinguishing responders to suvorexant from non-responders before starting treatment, which may help clinicians with decisions on optimal drug selection. Furthermore, the present study may demonstrate a role for the VTA and glia in the orexin system. Thus, future studies and drug development considering the relation between glia in VTA and insomnia may be useful.

## Data Availability Statement

The data analyzed in this study is subject to the following licenses/restrictions: Clinical datasets of participants presented in this article are not readily available due to ethical reasons. Please contact minagaki@med.shimane-u.ac.jp for questions. Requests to access these datasets should be directed to Masatoshi Inagaki, minagaki@med.shimane-u.ac.jp.

## Ethics Statement

The studies involving human participants were reviewed and approved by the ethics committee of Shimane University Hospital. The patients/participants provided their written informed consent to participate in this study. Written informed consent was obtained from the individual(s) for the publication of any potentially identifiable images or data included in this article.

## Author Contributions

MIz: conceptualization, methodology, validation, data curation, formal analysis, investigation, and writing—original draft preparation. SM, KO, MH, and SH: writing—review and editing. MN and HK: supervision. HA: data curation, formal analysis, investigation, and writing—review and editing. MIn: writing—review and editing and supervision. All authors contributed to the article and approved the submitted version.

## Conflict of Interest

MIn received grants from the Research for Promotion of Cancer Control Programs during this study. He has also received lecture fees from Technomics, Fuji Keizai, Novartis, Yoshitomiyakuhin, Pfizer, MSD, Meiji Seika Pharma, Eisai, Otsuka, Sumitomo Dainippon Pharma, Mochida, Janssen, Takeda, and Eli Lilly. His institute has received research funds from Otsuka, Eisai, Daiichi-Sankyo, Pfizer, Astellas, MSD, Takeda, Fujifilm, Shionogi, and Mochida. HK has received lecture fees from Eisai, FUJIFILM Toyama Chemical Co., Ltd, Guerbet Japan, and Bayer. His institute has received research funds from Fuji Pharma Co., Ltd., Eisai, Guerbet Japan, Daiichi-Sankyo, Nihon Medi-Physics Co., Ltd., and FUJIFILM Toyama Chemical Co., Ltd. MIz has received lecture fee from Eisai. This study has NOT supported any institute including MSD, Merck unit in Japan, which sells suvorexant. The remaining authors declare that the research was conducted in the absence of any commercial or financial relationships that could be construed as a potential conflict of interest.

## Publisher's Note

All claims expressed in this article are solely those of the authors and do not necessarily represent those of their affiliated organizations, or those of the publisher, the editors and the reviewers. Any product that may be evaluated in this article, or claim that may be made by its manufacturer, is not guaranteed or endorsed by the publisher.

## Supplementary Material

Data Sheet 1 STROBE checklist.
