# Supplementary material for: Magnetic Resonance Spectroscopy in the Ventral Tegmental Area Distinguishes Responders to Suvorexant Prior to Treatment: A 4-Week Prospective Cohort Study
Source: Front Psychiatry. 2021 Aug 23;12:714376. doi: 10.3389/fpsyt.2021.714376 (PMC8419448; doi:10.3389/fpsyt.2021.714376)
Supplement: Data Sheet 1 — STROBE checklist. [file Data_Sheet_1.pdf]

Supplementary Material

Supplementary Table 1 Each component of PSQI before and after four-week suvorexant treatment for suvorexant responders and non-responders

	Before treatment		After treatment	
Component	Responders (n = 20)	Non-responders (n = 21)	Responders (n = 20)	Non-responders (n = 21)
Component 1:Subjective sleep quality	2(2-3)	1(1-2)	1(0.5-1.5)	2(1-3)
Component 2:Sleep latency	3(3-3)	2(1-3)	2(2-3)	3(2-3)
Component 3:Sleep duration	2(1.5-3)	1(0-2)	0.5(0-1)	2(0-3)
Component 4:Habitual sleep efficiency	2.5(1-3)	1(0-2)	1(0-1)	2(1-3)
Component 5:Sleep disturbances	1(1-2)	1(1-2)	1(1-1)	1(1-1)
Component 6:Use of sleeping medication	3(3-3)	3(2-3)	3(3-3)	3(3-3)
Component 7:Daytime dysfunction	2(1-2)	1(1-2)	0(0-1)	1(0-2)

Component 1(subjective sleep quality), component 2 (sleep latency), component 3 (sleep duration), component 4 (habitual sleep efficiency), and component 7 (daytime dysfunction) were decreased (improved) between before and after four-week suvorexant use among responders, while component 1(subjective sleep quality), component 2 (sleep latency), component 3 (sleep duration), component 4 (habitual sleep efficiency) were increased (worsened) among non-responders. PSQI: the Pittsburgh Sleep Quality Index. Data were presented as median (interquartile range).

Supplementary Table 2. The number of patients who use each drug category

	Before suvorexant use		Four weeks after suvorexant	
	responders	non-responders	responders	non-responders
The number of antidepressants users	8	4	7	2
The number of antiepileptic users	7	6	6	5
The number of antipsychotics users	13	11	12	13
The number of GABAergic users	13	12	9	6
The number of Ramelteon users	3	3	2	5

Antidepressants include amitriptyline, mianserin, trazodone, fluvoxamine, mirtazapine, duloxetine, escitalopram. Antiepileptics include phenytoin, fosphenytoin, carbamazepine, clonazepam, valproate, topiramate, lamotrigine, levetiracetam. Antipsychotics include chlorpromazine, perphenazine, levomepromazine, haloperidol, zotepine, risperidone, quetiapine, perospirone, olanzapine, aripiprazole, blonanserin, clozapine, paliperidone, asenapine, brexpiprazole. Gamma-aminobutyric acid (GABA) receptor agonists (GABAnergics) include estazolam, nitrazepam, flunitrazepam, brotizolamm, diazepam, lorazepam, tofisopam, loflazepate, flunitrazepam, midazolam, rilmazafone, tandospirone, zolpidem, eszopiclone, etizolam.

STROBE Statement—Checklist of items that should be included in reports of ***cohort studies***

	Item No	Recommendation	Comment	Page
**Title and abstract**	1	(*a*) Indicate the study's design with a commonly used term in the title or the abstract.	We added 'a 4-week prospective cohort study' to the title.	Title, page 1.
		(*b*) Provide in the abstract an informative and balanced summary of what was done and what was found.	The abstract is structured. What was done is stated in the methods and what was found is stated in the results and conclusions.	Abstract, page2.
**Introduction**				
Background/ rationale	2	Explain the scientific background and rationale for the investigation being reported.	We explained the scientific background and rationale for the investigation in the introduction.	Introduction (page 3).
Objectives	3	State specific objectives, including any prespecified hypotheses.	We stated our hypotheses in the third paragraph of the introduction.	The third paragraph of Introduction (page 3).
**Methods**				
Study design	4	Present key elements of study design early in the paper.	We state 'The present 4-week cohort study' in the second paragraph of the methods, 'measurement' in the third paragraph of the methods, and 'MRS' in the fifth paragraph of the methods.	Methods (page 3)).
Setting	5	Describe the setting, locations, and relevant dates, including periods of recruitment, exposure, follow-up, and data collection.	We reported these data in the Participants section of the methods.	'Participants' section of methods (page 3).
Participants	6	(*a*) Give the eligibility criteria, and the sources and	Inclusion criteria are described in the Participants section of	'Participants' section in

		methods of selection of participants. Describe methods of follow-up.	the methods. We observed patients for 4 weeks of suvorexant use as described in the ‘measurement’ section of the methods.	methods (page 4) and ‘measurement’ section in methods (page 4).
		(*b*) For matched studies, give matching criteria and number of exposed and unexposed.	No matching was performed.	
Variables	7	Clearly define all outcomes, exposures, predictors, potential confounders, and effect modifiers. Give diagnostic criteria, if applicable.	All data are clearly described in the methods.	Methods (page 3).
Data sources/ measurement	8*	For each variable of interest, give sources of data and details of methods of assessment (measurement). Describe comparability of assessment methods if there is more than one group.	Sources of data are described in the methods, Table 1, Table 2, and Table 3.	Methods (page 3), Table 1, Table 2, and Table 3.
Bias	9	Describe any efforts to address potential sources of bias.	Potential sources of bias were discussed in the last paragraph of Discussion.	Last paragraph of discussion (page 10).
Study size	10	Explain how the study size was arrived at.	Calculation of the study size was stated in the ‘statistical analysis’ section of the methods.	Statistical analysis (page 6).
Quantitative variables	11	Explain how quantitative variables were handled in the analyses. If applicable, describe which groupings were chosen and why.	Quantitative variables were reported using the median and interquartile range.	Statistical analysis (page 6), Table 1, Table 2, and Table 3.
Statistical methods	12	(*a*) Describe all statistical methods, including those	Statistical methods were described in the statistics section. We performed the	Statistical analysis (page 6).

		used to control for confounding.	Mann–Whitney *U* test for magnetic resonance spectroscopy and the Kruskal–Wallis test for drug data.	
		(*b*) Describe any methods used to examine subgroups and interactions.	No subgroup examination was performed.	Statistical analysis (page 6).
		(*c*) Explain how missing data were addressed.	We conducted a full case analysis and patients who lacked data were excluded.	Methods (page 3).
		(*d*) If applicable, explain how loss to follow-up was addressed.	Patients with missing data were excluded.	Methods (page 3).
		(*e*) Describe any sensitivity analyses.	No sensitivity analyze was performed.	Methods (page 3).
**Results**				
Participants	13*	(a) Report numbers of individuals at each stage of study—e.g., numbers potentially eligible, examined for eligibility, confirmed eligible, included in the study, completing follow-up, and analyzed.	The numbers of individuals were reported in the first paragraph of the results and in Figure 2.	The first paragraph of the Results (page 7) and Figure 2.
		(b) Give reasons for non-participation at each stage.	The exclusion criteria are described in the participants in the methods and in Figure 2.	Participants (page 3) and Figure 2.
		(c) Consider use of a flow diagram.	Figure 2 is a flow diagram.	Figure 2.
Descriptive data	14*	(a) Give characteristics of study participants (e.g., demographic, clinical, social) and information on exposures and potential confounders.	The characteristics of study participants are shown in Table 1.	Table 1.
		(b) Indicate number of participants with missing	Excluded patients are described in the first	The first paragraph of the

		data for each variable of interest.	paragraph of the results and in Figure 2.	Results (page 7) and Figure 2.
		(c) Summarize follow-up time (e.g., average and total amount).	The follow-up time was 4 weeks of suvorexant use.	Methods (page 6).
Outcome data	15*	Report numbers of outcome events or summary measures over time.	Outcomes are described in results, Table 1, and Figure 3.	Results (page 7), Table 1, and Figure 3.
Main results	16	(*a*) Give unadjusted estimates and, if applicable, confounder-adjusted estimates and their precision (e.g., 95% confidence interval). Make clear which confounders were adjusted for and why they were included.	Primary outcomes are described in the third paragraph of the results and Figure 3. No confounder-adjusted estimates were performed.	The third paragraph of the Results (page 8) and Figure 3.
		(*b*) Report category boundaries when continuous variables were categorized.	No continuous variables were categorized.	
		(*c*) If relevant, consider translating estimates of relative risk into absolute risk for a meaningful time period.	No relevant analysis was conducted.	
Other analyses	17	Report other analyses done—e.g., analyses of subgroups and interactions, and sensitivity analyses.	The choline/creatinine ratio in the ventral tegmental area between responders and non-responders stratified by each diagnostic criteria of comorbid psychiatry disease was reported in Table 4.	Table 4
**Discussion**				
Key results	18	Summarize key results with reference to study objectives.	We summarized our key results in the first paragraph of the discussion.	The first paragraph of Discussion (page 8).

Limitations	19	Discuss limitations of the study, taking into account sources of potential bias or imprecision. Discuss both direction and magnitude of any potential bias.	We discussed limitations in the fifth paragraph of the discussion.	The fifth paragraph of discussion (page 9).
Interpretation	20	Give a cautious overall interpretation of results considering objectives, limitations, multiplicity of analyses, results from similar studies, and other relevant evidence.	We cautiously interpreted our results in the discussion.	Discussion (page 8).
Generalizability	21	Discuss the generalizability (external validity) of the study results.	The generalizability is discussed in each paragraph of the discussion.	Discussion (page 8).
**Other information**				
Funding	22	Give the source of funding and the role of the funders for the present study and, if applicable, for the original study on which the present article is based.	Funding is described in the conflict of interest section.	Conflict of interest (page 12).

*Give information separately for exposed and unexposed groups.

Note: An Explanation and Elaboration article discusses each checklist item and gives methodological background and published examples of transparent reporting. The STROBE checklist is best used in conjunction with this article (freely available on the Web sites of PLoS Medicine at http://www.plosmedicine.org/, Annals of Internal Medicine at http://www.annals.org/, and Epidemiology at http://www.epidem.com/). Information on the STROBE Initiative is available at http://www.strobe-statement.org.
